# Successful Laparoscopic Hepaticojejunostomy for Infant Congenital Biliary Dilatation with both Aberrant Right Hepatic Artery and Bile Duct from the Caudate Region

**DOI:** 10.1055/s-0044-1779624

**Published:** 2024-02-13

**Authors:** Shun Onishi, Koji Yamada, Masakazu Murakami, Toshio Harumatsu, Takafumi Kawano, Satoshi Ieiri

**Affiliations:** 1 Department of Pediatric Surgery, Research Field in Medical and Health Sciences, Medical and Dental Area, Research and Education Assembly, Kagoshima University, Sakuragaoka, Kagoshima City, Japan

**Keywords:** congenital biliary dilatation, laparoscopic surgery, aberrant hepatic artery, aberrant hepatic duct

## Abstract

A boy with congenital hydronephrosis underwent ultrasonography every month for follow-up. At 4 months of age, ultrasonography incidentally revealed congenital biliary dilatation (5-cm type Ia). We performed laparoscopic extrahepatic bile duct resection and hepaticojejunostomy. After dissecting the dilated common bile duct (CBD), we found that the arcading-like shaped right hepatic artery (RHA) coursed in front of the CBD. Additionally, a tiny duct was identified below the main hepatic duct. At first, we thought it was a lymphatic vessel and dissected it from the main hepatic duct. However, bile flow out was recognized after dissecting the tiny duct. Finally, we confirmed it as an aberrant bile duct from the caudate region. We anastomosed the bile duct from the caudate region and main hepatic duct in a double-barrel fashion and performed hepaticojejunostomy below the RHA. The postoperative course was uneventful. Ultrasonography showed no intrahepatic ductal dilatation including the caudate lobe.

## Introduction

A definitive preoperative diagnosis of aberrant vessels and bile ducts in congenital biliary dilatation is difficult in pediatric patients. 1 2 3 Aberrant vessels may lead to increased perioperative complications in hepatobiliary surgery. In pancreatoduodenectomy, for example, an aberrant right hepatic artery (RHA) may increase operative time, intraoperative blood loss, intraoperative/postoperative complications, and length of stay. 4 However, aberrant vessels in pediatric congenital biliary dilatation have not been discussed. We report a case of successful laparoscopic hepaticojejunostomy for a patient with an aberrant RHA and aberrant bile duct from the caudate region.

## Case Presentation



**Video 1** The video shows the operative procedure of extrahepatic bile duct resection and hepaticojejunostomy.


**Fig. 1:**
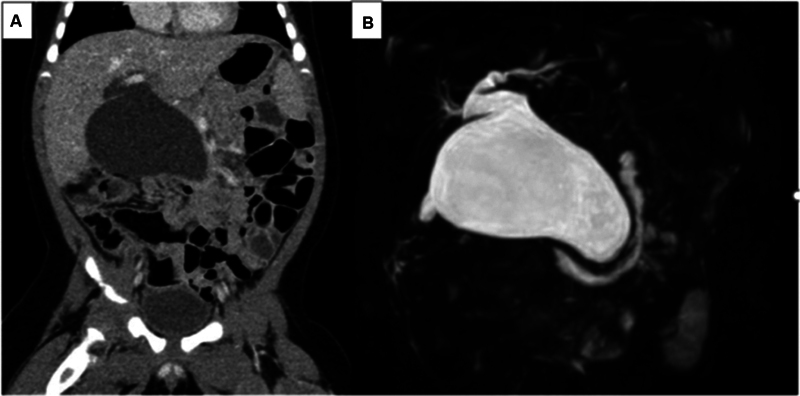
Computerized tomography ( **A** ) and magnetic resonance cholangiopancreatography ( **B** ) showed congenital biliary dilatation (5-cm type Ia [Todani classification]).

The patient was a boy with congenital hydronephrosis who had undergone ultrasonography every month for follow-up. At 4 months of age, ultrasonography incidentally showed an intra-abdominal cyst. Computerized tomography (CT) and magnetic resonance cholangiopancreatography revealed congenital biliary dilatation (5-cm type Ia [Todani classification]) ( Fig. 1A, B ). A definitive pancreaticobiliary maljunction and bile duct anomaly were not detected preoperatively. At 7 months of age, we performed extrahepatic bile duct resection and hepaticojejunostomy with a five-port layout.

Under general anesthesia, the patient was placed in a broad base position, and a 10-mm 30° laparoscope was inserted through an umbilical incision with a wound retractor. Pneumoperitoneum was established with 8 mm Hg CO _2_ inflation. Four additional trocars were inserted in the right upper abdomen (operator's left hand, 3.5-mm), right side of the umbilicus (operator's right hand, 5-mm), left side of the umbilicus (assistant's left hand, 3.5-mm), and left upper abdomen (assistant's right hand, 2.4-mm needle device).

**Fig. 2:**
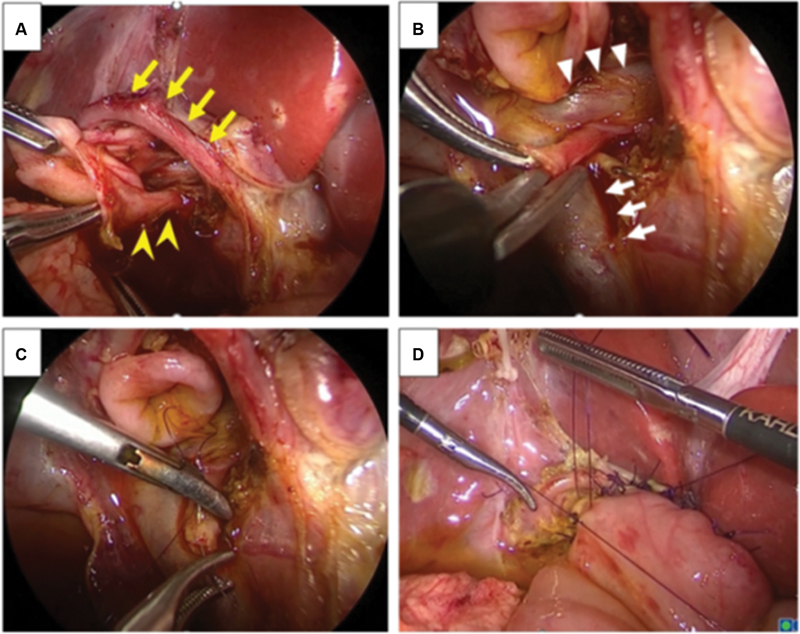
( **A** ) An aberrant right hepatic artery (RHA) coursed across the front of the common bile duct (yellow arrows). A tiny duct was identified next to the main hepatic duct (yellow arrowheads). ( **B** ) Bile flow was recognized from the tiny duct after dissecting the duct. The main portal vein (white arrows) and left portal vein (white arrowheads) were detected. ( **C** ) The bile duct from the caudate region was anastomosed to the main hepatic duct in a double-barrel fashion using 6-0 absorbable suture. ( **D** ) Hepaticojejunostomy was performed using interrupted intracorporeal knot tying.

After dissecting the dilated common bile duct (CBD), we found that the arcading-like shaped RHA coursed in front of the CBD ( Fig. 2A , Video 1 ). In addition, a tiny duct was identified below the main hepatic duct ( Fig. 2A ). We initially thought it was a lymphatic vessel and dissected it from the main hepatic duct ( Fig. 2B , Video 1 ). However, bile flow from the tiny duct was recognized after dissection. Finally, we confirmed it was an aberrant bile duct from the caudate region. The bile duct from the caudate region was anastomosed to the main hepatic duct in a double-barrel fashion using 6-0 absorbable suture ( Fig. 2C , Video 1 ).

The jejunum was extracted from the umbilical wound, and Roux-en Y jejunojejunostomy was performed. The mucosa and serosa of the opened hole were approximated using 6-0 absorbable suture to secure hepaticojejunostomy. The jejunum was pulled up through the retrocolic. Hepaticojejunostomy was performed below the aberrant RHA without repositioning the dorsal side of the anastomotic site due to the risk that it would compress the repositioned aberrant RHA. Anastomosis was performed without stent insertion ( Fig. 2D , Video 1 ).

The postoperative course was uneventful, without elevation of serum bilirubin. Ultrasonography showed no intrahepatic ductal dilatation, and hepatobiliary scintigraphy showed no biliary leaks or obstruction of hepaticojejunostomy. The patient was discharged on postoperative day 15. There were no intrahepatic ductal dilation and bilirubin elevation after 2 years.

## Discussion

Our group has reported several techniques for laparoscopic hepaticojejunostomy in pediatric patients. 5 6 In our institution, we standardized the following methods: (1) enlarge the small hepatic duct using a diagonal cut up the left side of the hepatic duct, (2) make an anastomotic hole at the anterior jejunal wall based on the hepatic duct size, and (3) approximate the mucosa and serosa of the opened hole to perform membrane-to-membrane anastomosis. These tips are handy for confirming the hepatic duct lumen.

There are no reports on whether aberrant RHA affect the perioperative outcome of pediatric congenital diaphragmatic hernia patients. Crocetti et al 4 reported that aberrant RHA increases the surgical complexity of pancreatoduodenectomy, negatively affecting intraoperative blood loss, length of operation, length in intensive care, and hospitalization, but does not influence long-term survival and disease-free rates.

Preoperative CT angiography imaging is beneficial to identify aberrant vessels. Chambers et al 7 showed a CT sensitivity, specificity, and accuracy of 96, 87, and 88%, respectively. We always evaluate enhanced CT scan and make three-dimensional image of vessels and bile duct. However, we could not detect the aberrant vessels in this case. Pediatric surgeons should always remember unexpected aberrant vessels that could not be identified preoperatively.

The aberrant RHA crossing in front of the hepatic duct is ordinarily repositioned on the dorsal side because it compresses the anastomotic site, leading to obstruction. However, the aberrant RHA in our case had a specific arcading shape; thus, it was considered that hepaticojejunostomy below the aberrant RHA would not affect the anastomotic site.
